# Comparing fiberoptic bronchoscopy and a tracheal tube-mounted camera-guided percutaneous dilatational tracheostomy: authors' reply 

**DOI:** 10.1186/s13054-018-2004-2

**Published:** 2018-03-23

**Authors:** Jörn Grensemann, Lars Eichler, Sophie Kähler, Dominik Jarczak, Marcel Simon, Hans O. Pinnschmidt, Stefan Kluge

**Affiliations:** 1 Department of Intensive Care Medicine, University Medical Center Hamburg-Eppendorf, Martinistraße 52, 20246 Hamburg, Germany; 2 Department of Respiratory Medicine, University Medical Center Hamburg-Eppendorf, Martinistraße 52, 20246 Hamburg, Germany; 3 Department of Medical Biometry and Epidemiology, University Medical Center Hamburg-Eppendorf, Martinistraße 52, 20246 Hamburg, Germany

We thank Xue et al. for their comments [1] regarding our article on the peri-interventional visualization of percutaneous dilatational tracheostomy (PDT) [2].

The authors noted that we did not provide diameters for the endotracheal tubes used in the bronchoscopy group. The endotracheal tubes had an inner diameter of 7.5 mm for female and 8.0 or 8.5 mm for male patients. Therefore, the differences between the inner tubes’ diameter and the 4.9 mm bronchoscopes used in our study were always ≥  2.0 mm as recommended [3] to maintain minute ventilation during bronchoscopy. The cross-sectional areas of endotracheal tubes with an inner diameter of 7.5, 8.0, and 8.5 mm are 44.2, 50.3, and 56.7 mm^2^, respectively. The effective cross-sectional area with a 4.9 mm bronchoscope inserted decreases to 25.3, 31.4, and 37.9 mm^2^ and to 36.6, 42.7, and 49.2 mm^2^ for a 3.1 mm bronchoscope, respectively. Although the decrease of the cross-sectional area is less pronounced when using a 3.1 mm bronchoscope potentially offering improved ventilation with less hypercarbia, it must be noted that the channel width of the Olympus LF-DP bronchoscope is only 1.2 mm compared to 2.2 mm in the Olympus BF-P60 used in our study. Regarding the respective cross-sectional areas of 1.1 and 3.8 mm^2^, it becomes clear that suctioning of secretions and especially blood from the trachea is nearly impossible with the smaller bronchoscope.

Concerning the fresh gas flow, we used critical care ventilators (Evita V500, Drägerwerk AG, Lübeck, Germany) with a semi-open breathing circuit. In semi-open breathing circuits, no rebreathing occurs and therefore the fresh gas flow may not play a role in hypercarbia compared to half-closed or closed anesthesia circuits that utilize a rebreathing circuit. In the ventilators used, the peak-inspiratory flow is controlled by the inspiratory ramp, which was set to 0 s in both groups, resulting in a peak-inspiratory flow of up to 180 L/min.

We chose to measure the procedure duration from skin incision to insertion of the tracheal cannula, since this duration is independent of variables such as time for fastening of the endotracheal tube or time of the surgical scrub disinfection that was done after changing the endotracheal tube in the VivaSight™-SL (VST) group. We agree with Xue et al. that our measurement does not reflect the overall time required for tracheostomy but allowed for a comparison of the secondary end-points hypercarbia and pH value.

Xue et al. conclude that bronchoscopy may offer several advantages, such as identification of the incision site. However, we could show that this is also identifiable by the VST. The loss of airway during tracheostomy without routine bronchoscopic guidance is estimated to be below 0.1% [4] and we doubt that routine bronchoscopy may further reduce this rare occurrence. Furthermore, the VST provides a view on the trachea and an accidental extubation can be readily identified.

We agree with Xue et al. that bronchoscopy has advantages over the VST as we had discussed in our article. Bronchoscopy allows for an inspection of the more distal parts of the trachea and also permits for immediate intervention should complications, especially bleeding, arise, confirmation of the correct position of the tracheal cannula after its insertion, and the clearance of blood and secretions from the bronchial tree. We also agree that bronchoscopy is currently the most frequently used technique for guidance of PDT [5], but it is not universally recommended due to the risk of hypercarbia [6]. Therefore, in selected patients, PDT with VST may offer the benefit of improved ventilation over bronchoscopy while retaining visual guidance.

## Abbreviations

PDT: Percutaneous dilatational tracheostomy
VST: VivaSight™-SL endotracheal tube

## References

[1] Xue FS, Wen C, Liu YY (2018). Comparing fiberoptic bronchoscopy- and a tracheal tube-mounted camera-guided percutaneous dilatational tracheostomy. Crit Care..

[2] Grensemann J, Eichler L, Kahler S, Jarczak D, Simon M, Pinnschmidt HO, Kluge S (2017). Bronchoscopy versus an endotracheal tube mounted camera for the peri-interventional visualization of percutaneous dilatational tracheostomy--a prospective, randomized trial (VivaPDT). Crit Care..

[3] Lawson RW, Peters JI, Shelledy DC (2000). Effects of fiberoptic bronchoscopy during mechanical ventilation in a lung model. Chest..

[4] Dennis BM, Eckert MJ, Gunter OL, Morris JA, May AK (2013). Safety of bedside percutaneous tracheostomy in the critically ill: evaluation of more than 3,000 procedures. J Am Coll Surg..

[5] Vargas M, Sutherasan Y, Antonelli M, Brunetti I, Corcione A, Laffey JG, Putensen C, Servillo G, Pelosi P (2015). Tracheostomy procedures in the intensive care unit: an international survey. Crit Care.

[6] Australian and New Zealand Intensive Care Society. Percutaneous dilatational tracheostomy: Consensus statement. 2014. http://www.anzics.com.au/Downloads/2014%20The%20ANZICS%20Percutaneous%20Dilatational%20Tracheostomy%20Consensus%20Statement.pdf.

